# The Evaluation of Dynamic FDG-PET for Detecting Epileptic Foci and Analyzing Reduced Glucose Phosphorylation in Refractory Epilepsy

**DOI:** 10.3389/fnins.2018.00993

**Published:** 2019-01-09

**Authors:** Yongxiang Tang, Jeih-San Liow, Zhimin Zhang, Jian Li, Tingting Long, Yulai Li, Beisha Tang, Shuo Hu

**Affiliations:** ^1^Department of PET Center, Xiangya Hospital Central South University, Changsha, China; ^2^Molecular Imaging Branch, National Institute of Mental Health, Bethesda, MD, United States; ^3^Department of Blood Transfusion, Xiangya Hospital Central South University, Changsha, China; ^4^National Clinical Research Center for Geriatric Diseases, Xiangya Hospital Central South University, Changsha, China

**Keywords:** epilepsy, PET imaging, dynamic imaging, kinetic modeling, glucose phosphorylation

## Abstract

**Aims:** Static fluorodeoxyglucose (FDG)-positron emission tomographic (PET) imaging plays an important role in the localization of epileptic foci. Dynamic FDG PET allows calculation of kinetic parameters. The aim of this study was to investigate whether kinetic parameters have potential for identifying epileptic foci, and to assess the correlation of parameters asymmetry indexes (ASYM) between dynamic and static FDG PET for understanding the pathophysiology of hypometabolism within intractable epilepsy.

**Methods:** Seventeen patients who had refractory epilepsy correctly localized by static FDG PET with good outcome after foci resection were included. Eight controls were also studied. We performed dynamic and static FDG PET scan before operation. Images of both scans were coregistered to the montreal neurological institute space, regional time activity curves and activity concentration (AC) were obtained by applying the automated anatomical labeling template to the two spatially normalized images, respectively. Kinetic parameters were obtained using a two-tissue non-reversible compartmental model with an image-derived input function. AC from the static scan was used. Side-to-side ASYM of both static AC and kinetic parameters were calculated and analyzed in the hypometabolic epileptogenic regions and non-epileptogenic regions.

**Results:** Higher values of ASYM from both kinetic parameters and static AC were found in the patients compared to the controls from epileptogenic regions. In the non-epileptogenic regions, no ASYM differences were seen between patients and controls for all parameters. In patients, static AC showed larger ASYM than influx (*K*_1_) and efflux (*k*_2_) of capillaries, but there were no statistical differences of ASYM between net metabolic flux (*K*_i_) or the phosphorylation (*k*_3_) and static AC. ASYM of static AC positively correlated with ASYM of *k*_3_.

**Conclusion:** Dynamic FDG PET can provide equally effective in detecting the epileptic foci compared to static FDG PET in this small cohort. In addition, compared to capillary influx, the hypometabolism of epileptic foci may be related to reduced glucose phosphorylation.

## Introduction

Epilepsy is considered as one of the most common chronic neurological diseases affecting 65 million people of all ages worldwide (Moshe et al., 2015). Interictal positron emission tomography (PET) measurements of glucose metabolism using fluorodeoxyglucose (FDG) has become a powerful diagnostic tool for presurgical delineation of patients with intractable epilepsy (Burneo et al., 2015), it demonstrated a sensitivity of 70–95% in patients with temporal lobe epilepsy (TLE) and 20–70% in extra-TLE patients, the highest clinical benefit of FDG PET can be achieved in patients with magnetic resonance imaging (MRI) negative TLE (Uijl et al., 2007; Muhlhofer et al., 2017; Sidhu et al., 2018). Studies have shown that FDG PET has greatly improved epilepsy patient’s outcome probably by better identifying and understanding the hypometabolism epileptogenic zone (Zhu et al., 2017).

Hypometabolism of epileptogenic focus delineation for surgical planning by using FDG PET is classically based on static images (Engel et al., 1990; Wieser and ILAE Commission on Neurosurgery of Epilepsy, 2004; Uijl et al., 2007). Interpretation of FDG PET image usually involves assessing left-right asymmetries in activity concentration (AC) or standardized uptake value (SUV). Static AC or SUV from the static maps had been demonstrated to be useful for locating epileptic foci in many studies, but these findings have not been consistent (Engel et al., 1990; Wieser and ILAE Commission on Neurosurgery of Epilepsy, 2004; Uijl et al., 2007). Static AC or SUV is calculated based on the FDG concentration of a region at a single time point normalized by the administered FDG activity and body weight, it is a relative measurement and can be affected by various factors (Devriese et al., 2016). Absolute quantitative method using dynamic PET scan was applied to study the pattern of FDG metabolism in brain (Huang et al., 2015). Quantitative analysis of dynamic FDG PET studies may provide more information than a single time point measurement for the epileptic region and potentially improving the diagnosis and treatment of patients.

These methodologies, first developed by Phelps et al. (1979) for studying cerebral metabolism, have been applied to other tissues and have been suggested to be more reliable in reflecting glucose metabolism than the conventional method of using static AC or SUV for quantitation. Kinetic parameters of FDG metabolism can be calculated by using dynamic FDG PET (Vriens et al., 2010). It has been shown that kinetic parameters analysis was helpful for diagnosis of central nervous system lymphoma and for differentiation between high-grade glioma and CNS lymphoma (Nishiyama et al., 2007; Kimura et al., 2009), kinetic parameters maps have an increased signal-to-background ratio relative to static AC or SUV maps because the free FDG in the background was teased out by the model (Visser et al., 2008). Therefore, we hypothesized that interictal side-to-side asymmetry in epilepsy patients by using dynamic FDG PET may have an important capacity as static FDG PET for detecting epileptic region.

Additionally, one of the most persistent problems is the unknown pathophysiology of interictal hypometabolism in the area related to epilepsies (Henry, 1996; Jupp et al., 2012). By using dynamic FDG PET, distinction can be made between unmetabolized FDG and bound FDG-6-phosphate which may provide more insight into the glucose metabolic rate and metabolic vascular heterogeneity of epileptic foci. This biological information is likely to be relevant for uncovering the mechanism of epileptic hypometabolism, further benefit understanding and treatment of epilepsy.

We studied patients who had medically intractable epilepsy correctly localized by static FDG PET with good outcomes after seizure foci resection were enrolled, with age-matched controls. The aim of this study was to investigate whether kinetic parameters have capacity for identifying epileptic foci in epilepsy evaluation. Specifically, we assess the correlation of ipsilateral and contralateral asymmetry indexes (ASYM) between kinetic parameters from dynamic FDG PET and static AC from static FDG PET in an effort to try to understand the underline mechanism of the hypometabolism within epileptic foci.

## Materials and Methods

This study was carried out in accordance with the recommendations of Commission of Medical Research Involving Human Subjects at Region of Xiangya Hospital with written informed consent from all subjects. All subjects gave written informed consent in accordance with the Declaration of Helsinki. The protocol was approved by the Commission of Medical Research Involving Human Subjects at Region of Xiangya Hospital, Central South University, China.

### Patient Population

Seventeen patients with drug-resistant epilepsy were studied. Patients were evaluated with static FDG PET scans as well as MRI with T1-weighted and T2-weighted sequences. For presurgical evaluation of each patient, at least 2 experienced epileptologists participated the process to determine the location of the epileptogenic zone based on all available clinical, video-electroencephalographic, imaging, and neuropsychologic data. When the study results were discrepant, the surgical sites were determined by the invasive EEG studies. Then patients were referred to the clinical epilepsy section. All 17 had epileptic foci correctly localized by static FDG PET before operation with good outcomes (followed for at least 1 year, Engel class I or II) after seizure foci resection. Epileptic foci were well-localized in temporal lobe (*n* = 11), frontal lobe (*n* = 2), occipital lobe (*n* = 2), parietal lobe (*n* = 1), and insular lobe (*n* = 1) with concordant static FDG hypometabolism (based on routine clinical reports). Table 1 detailed the demographics and clinical features of the patients. Patients were excluded if they had medical illness with central nervous system impact other than epilepsy, e.g., head trauma, encephalitis, tumor, infarct, or porencephaly, as well as those with previous resection surgery within 1 year postoperatively.

**Table 1:** Summary of patient’s demographic and clinical characteristics enrolled in study.

Characteristic	Patients
Patients	17
Median age (range)	21 (11–33)
**Focus by FDG PET and Seizure Focus Resection (%)**	
Frontal lobe	2 (12)
Parietal lobe	1 (6)
Temporal lobe	11 (64)
Occipital lobe	2 (12)
Insular lobe	1 (6)
**Histopathology (%)**	
Focal cortical dysplasia	6 (35)
Mesial temporal sclerosis	11 (65)
**Engel Epilepsy Surgery Outcome Scale (%)**	
Class I: Free of disabling seizures	15 (88)
Class II: Rare disabling seizures (“almost seizure-free”)	2 (12)

To exclude possible physiological left-right asymmetry. Eight age-matched (median age, 26 years; age range, 15–34 years) controls were also studied. All volunteers had no abnormal findings on MRI, had no history of neurologic diseases, psychologic diseases, or severe medical illnesses; and were taking no drugs known to affect brain FDG uptake.

### FDG PET Image Acquisition and Processing

Fluorodeoxyglucose PET scans were performed at the PET Center of Xiangya Hospital on the Discovery Elite PET/CT scanner (GE Healthcare) before the surgical resection. Images were acquired in three dimensions. The full width at half maximum of the scan was 5.4 mm. All images were reconstructed into a, 128 × 128 *trans*-axial matrix (field of view of 35 cm) using the 3D VUE point (GE Healthcare) ordered-subset expectation maximization algorithm with six iterations, six subsets. A low dose CT scan was obtained simultaneously for attenuation correction.

The FDG was prepared according to the method described by O’Brien et al. (2008). The radiochemical purity was greater than 97%. Patients fasted 4–6 h before FDG injection and rested in a quiet room, with the head positioned comfortably on the table and immobilized with tape. Dynamic FDG PET scan 60-min started immediately after a bolus injection of FDG (3.7 MBq/kg) via the left antecubital vein, with no arterial blood sampling. Images were reconstructed into 39 frames of 12 × 5 s, 6 × 10 s, 6 × 30 s, 6 × 120 s, 10 × 300 s. Patients also underwent a static FDG PET scan after dynamic scan as part of their routine clinical investigation.

Positron emission tomographic imaging procedure and the post-imaging interview were video monitored to ensure patient’s safety and the quality of scan acquisition, e.g., to see if there was a seizure or movement during the study course. Dynamic and static FDG PET scans for the eight healthy controls were also obtained following the same procedure.

### Blind Visual Assessment for Static FDG PET Images

Two experienced nuclear medicine physicians, blind to clinical data and imaging results, independently assessed the regional metabolism. The most hypometabolic region on the static FDG PET scan was determined to be the epileptogenic zone. If interpretation differed, a consensus opinion was reached after joint review.

### Treatment and Follow-Up

The extent of the resection was determined according to all available clinical information, when the study results were discrepant, the surgical sites were determined by the invasive subdural ictal EEG findings. 11 patients had radical temporal lobectomy, 2 patients had standard frontal lobectomy, 2 patients had partial occipital lobectomy, 1 patient had partial parietal lobectomy, and 1 patient had radical insular lobectomy. Fifteen patients were seizure free (Engel class I: Free of disabling seizures), and 2 patients had rare seizures during the follow-up after surgery (Engel class II: Rare disabling seizures, “almost seizure-free”).

### PET Image Analysis

**FIGURE 1:**
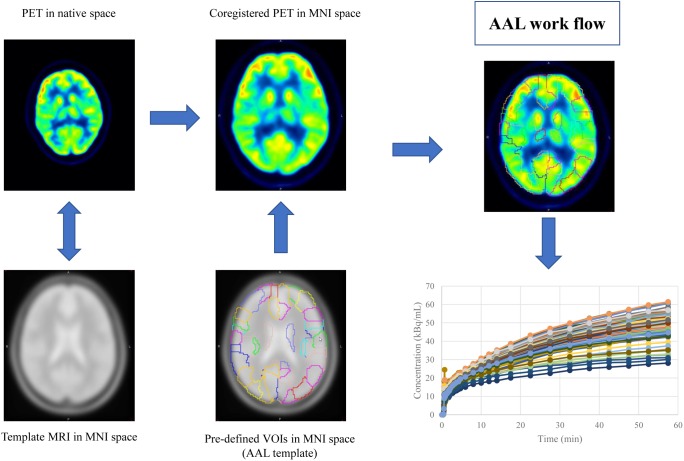
The work flow of spatial normalization. Dynamic or static PET image were co-registered to the spatially normalized single-subject high-resolution T1 volume provided by the Montreal Neurological Institute (MNI) space. The transformation information was then used to reorient the image into a standardized space and further used as landmarks for the 3D definition of 45 anatomical volumes of interest (AVOI) in each hemisphere. 90 AVOI were reconstructed and assigned with a label individually (performed Automated Anatomical Labeling, AAL). Regional time activity curves and radioactivity concentration were obtained by applying the AAL template to the two spatially normalized images, respectively.

Mean image of the dynamic scan was co-registered to an MRI template in the Montreal Neurological Institute (MNI) space. The transformation information was then used to reorient the dynamic image into the MNI space. The same process was also applied to the static image. Regional time activity curves and radioactivity concentration were obtained by applying a set of predefined volumes of interest (VOIs) [AAL template (Tzourio-Mazoyer et al., 2002)] to the two spatially normalized images, respectively (Figure 1).

**FIGURE 2:**
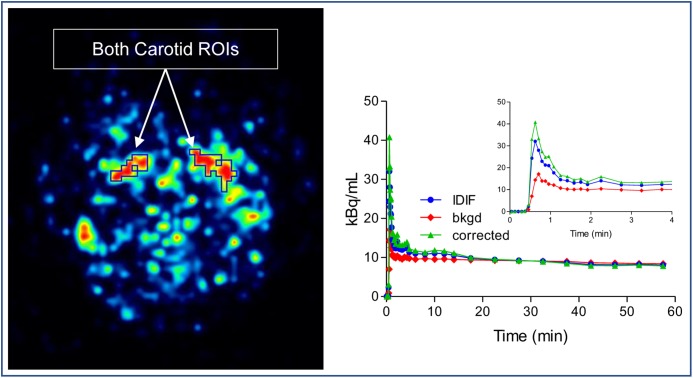
Image derived input function (IDIF) extraction method. Both left and right carotid regions of interest (ROI) were drawn on slices in the early frames of the dynamic image and an immediate background ROI surrounding the carotid ROI was also drawn. The corrected IDIF can be calculated by model equation.

As an alternative to arterial sampling, image derived input function (IDIF) was utilized as described by Zanotti-Fregonara et al. (2011). In brief, the method was followed but with no blood scaling: both left and right carotid regions of interest (ROI) were manually drawn on two consecutive slices in the early frames of the dynamic image. For each carotid ROI, an immediate background ROI surrounding the carotid ROI was also drawn. Based on the model equation below:

$$C_{\text{meas}}(t)=\mathrm{RC}\times C_{\text{input}}(t)+\mathrm{SP}\times C_{\text{bkgd}}(t)$$

where *C*_meas_ indicate the measured carotid concentration of radioactivity from the carotid ROI. *C*_bkgd_ represent the concentration of radioactivity from the background ROI. RC is the recovery coefficient, SP is the spill in factor from the surrounding. *C*_input_ can be calculated. Because there were no blood measurements, both RC and SP were estimated from simulation based on the reconstructed image resolution from GE Discovery. RC and SP varied slightly among subjects depending on the size and shape of their ROIs (Figure 2).

From the dynamic PET measurements, kinetic parameters were calculated using a two-tissue irreversible compartmental model with brain time activity curves and the input function (PMOD 3.1, PMOD Technologies Ltd.). rate constants of FDG influx (*K*_1_), efflux (*k*_2_), phosphorylation (*k*_3_) were determined for each patient, and net metabolic flux (*K*_i_) representing the net rate of FDG consumption by the cerebral can be calculated by *K*_i_ = (*K*_1_ × *k*_3_)/(*k*_2_ + *k*_3_). From their static PET, static AC was used. These rate constants in each epileptogenic focus were compared with its contralateral VOIs. For each parameter, paired analysis of asymmetry index between the ipsilateral and contralateral VOIs was computed.

**FIGURE 3:**
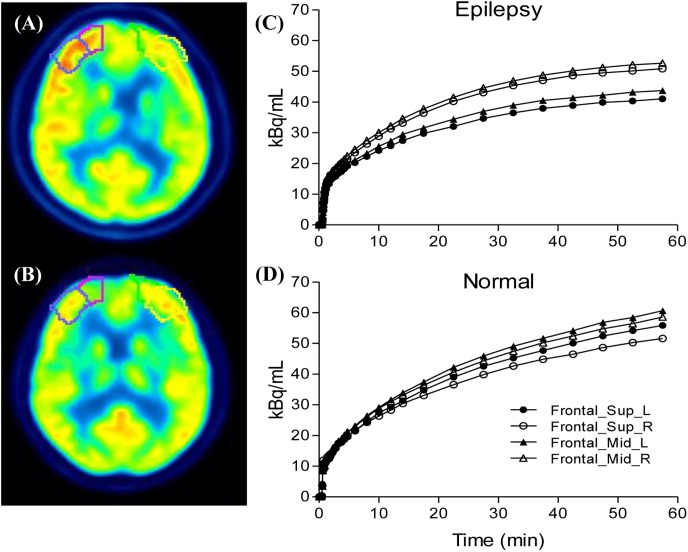
Compare the ipsilateral and contralateral regions between patients and controls: Illustration of drawn region of interest **(A,B)**, the processes were performed for both the epileptogenic foci and the counterpart homologous regions in the contralateral hemispheres. Generated time activity curves **(C,D)**, dynamic FDG PET kinetic parameters and static FDG PET activity concentration of asymmetry index can be calculated.

We selected epileptogenic VOIs from AAL template inside the extent of epileptogenic foci, which were determined by regions of resection surgery and hypometabolic zone of static FDG PET, if the surgical regions are large and include multiple brain regions, we selected only one predefined VOI inside this region which had most reduced FDG concentration in the static maps. A total of six epileptogenic VOIs in five brain lobes were selected from 17 patients, including Hippocampus (*n* = 11), Occipital_Inf (*n* = 2), Frontal_Sup and_Mid (*n* = 1), Frontal_Inf_Orb (*n* = 1), SupraMarginal and Angular (*n* = 1), Insula (*n* = 1). The background VOIs at the counterpart homologous regions was selected for each patient. Asymmetry index [*ASYM* = (contralateral − ipsilateral) × 2/(contralateral + ipsilateral)] was calculated. To exclude physiological asymmetry, six VOIs mentioned above were selected from control group. Absolute value of left/right ASYM were calculated using the same equation, and six ASYMs were combined and calculated the average ASYM for each control (Figure 3). Taking into account the possible effects of image noise, we selected the same non-epileptic VOIs in the four brain regions (Frontal, Parietal, Occipital, and Temporal) of the patients and the controls to calculate the average asymmetry index as a reference, these VOIs are relatively large, including Frontal_Sup_Medial/Mid_Orb, Precentral/Parietal_Sup/Parietal _Inf, Occipital_Sup/_Mid, Temporal_Sup/_Mid/_Inf.

### Statistical Analysis

All data were analyzed by the SPSS software (IBM SPSS Statistics, Version 18.0). Data were summarized as mean. Differences between ASYM of epileptogenic and non-epileptogenic regions were quantified by using the Wilcoxon rank test and the reductions were reported in parameter values. To confirm the most observational interpretable Visual-based qualitative data, Wilcoxon rank tests were also carried out to compare ASYM of static AC with all ASYM of kinetic parameters. To understand the possible mechanism of the hypometabolism within epileptic foci, spearman correlation was used to determine associations between ASYM of static AC and ASYM of kinetic parameters (*K*_1_, *k*_2_, *k*_3_, and *K*_i_). A *P*-value less than 0.05 (*P* < 0.05) was considered statistically significant.

## Results

After carefully reviewing EEG, MRI, PET results, medical records of seizure foci resection and clinical follow-up, a total of 17 patients were included in this study. At the regions of epileptic foci, estimated asymmetry index for *K*_1_, *k*_2_, *k*_3_, *K*_i_ and static AC of the 17 patients averaged 0.12, 0.14, 0.21, 0.27, and 0.24, for controls at all the corresponding regions, averaged asymmetry indexes of *K*_1_, *k*_2_, *k*_3_, *K*_i_ and static AC were 0.002, 0.003, 0.004, 0.003, and 0.004. The ASYM of *K*_1_, *k*_2_, *k*_3_, *K*_i_, static AC in the patients and controls non-epileptogenic regions were 0.04, 0.03, 0.05, 0.05, 0.05, and 0.03, 0.02, 0.06, 0.07, 0.06.

### Comparison of All ASYM Between Epileptogenic and Non-epileptogenic Regions

**FIGURE 4:**
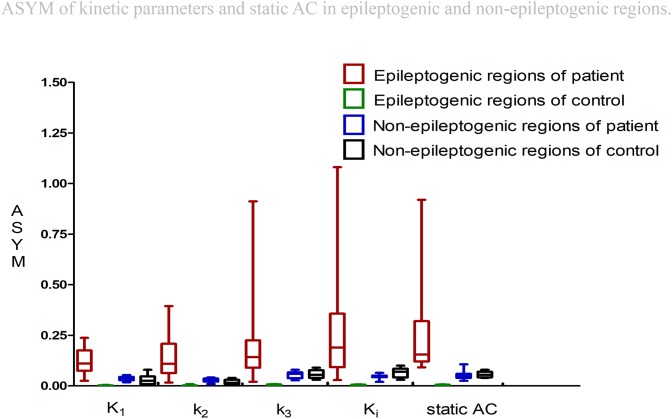
Asymmetry indexes (ASYM) of kinetic parameters and static AC in epileptogenic and non-epileptogenic regions. In epileptogenic regions, the estimated mean ASYM in controls at all corresponding regions averaged 0.002 for *K*_l_, 0.003 for *k*_2_, 0.004 for *k*_3_, 0.003 for *K*_i_, and 0.004 for static AC, higher values of ASYM from both kinetic parameters and static AC were found in the patients compared to the controls (Wilcoxon rank test, all *P* < 0.001). In non-epileptogenic regions, the ASYM of *K*_1_, *k*_2_, *k*_3_, *K*_i_, static AC in the patients and controls non-epileptogenic regions were 0.04, 0.03, 0.05, 0.05, 0.05 and 0.03, 0.02, 0.06, 0.07, 0.06. No ASYM differences were observed in non-epileptogenic regions between patients and controls for every parameter (Wilcoxon rank test, all *P* > 0.05). In patients, static AC showed more variability than *K*_1_ and *k*_2_ (mean ASYM of static AC = 0.24, *k*_2_ = 0.14, *K*_1_ = 0.12; Wilcoxon rank test, both *P* < 0.05), but there were no statistical differences among *K*_i_, *k*_3_, static AC (mean ASYM of *K*_i_ = 0.27, *k*_3_ = 0.21; Wilcoxon rank test, all *P* > 0.2).

All ASYM from patients and controls were illustrated in Figure 4. For epileptogenic regions, higher values of ASYM from both kinetic parameters and static AC were found in the patients compared to the controls (Wilcoxon rank test, all *P* < 0.001). Each parameter was significantly reduced in the seizure foci compared with that in contralateral healthy hemispheres. No ASYM differences were seen in non-epileptogenic regions between patients and controls for every parameter (Wilcoxon rank test, all *P* > 0.05). But ASYMs in non-epileptogenic regions of patients and controls are all bigger than healthy controls in regions corresponding to patient’s all the selective AAL VOIs from epileptogenic foci (Wilcoxon rank test, all *P* < 0.001). The selection range of our epileptogenic VOI was small, and the selection range of non-epileptic areas was relatively large. Larger ASYM of the non-epileptic area of the patient and control than the small control area corresponding to the epileptogenic regions may be attributed to greater biological variability in the brain. Despite statistical significance, visual assessment was not affected because the difference is small and not distinguishable. The ASYM of every parameter in our epileptic focus regions are significantly larger than the non-epileptogenic regions, which is the most needed for visual assessment.

### Visual Assessment and Comparing ASYM of Static AC and All ASYM of Kinetic Parameters in Patients

**FIGURE 5:**
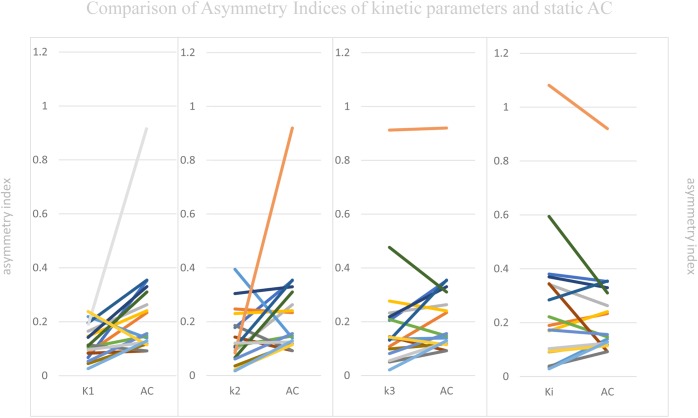
Comparison of Asymmetry Indices of kinetic parameters and static AC. Static AC showed a bigger difference than influx (*K*_1_) and efflux (*k*_2_) of capillaries (mean ASYM of static AC = 0.24, *k*_2_ = 0.14, *K*_1_ = 0.12; Wilcoxon rank test, both *P* < 0.05), but there were no statistical differences between net metabolic flux (*K*_i_) or the phosphorylation (*k*_3_) and static AC (mean ASYM of *K*_i_ = 0.27, *k*_3_ = 0.21; both *P* > 0.2).

Visual Assessment of hypometabolic epileptogenic regions, with parallel side-by-side view to show clear metabolic asymmetry. A further semiquantitative analysis, absolute ASYM was calculated to evaluate the intensity of the metabolic abnormality in brain regions. In general, the larger the ASYM, the easier it is to identify the hypometabolic epileptogenic regions. Comparison between different parameters in the patient’s epileptogenic VOI, static AC showed larger ASYM than influx (*K*_1_) and efflux (*k*_2_) of capillaries. This difference was statistically significant (mean ASYM of static AC = 0.24, *k*_2_ = 0.14, *K*_1_ = 0.12; Wilcoxon rank test, both *P* < 0.05), but there were no statistical differences of ASYM between net metabolic flux (*K*_i_) or the phosphorylation (*k*_3_) and static AC (mean ASYM of *K*_i_ = 0.27, *k*_3_ = 0.21; both *P* > 0.2). The reductions in *k*_3_ and *K*_i_ were similar to static AC seen in these patients (Figure 5).

### Correlation Between ASYM of Static AC and ASYM of Kinetic Parameters

**FIGURE 6:**
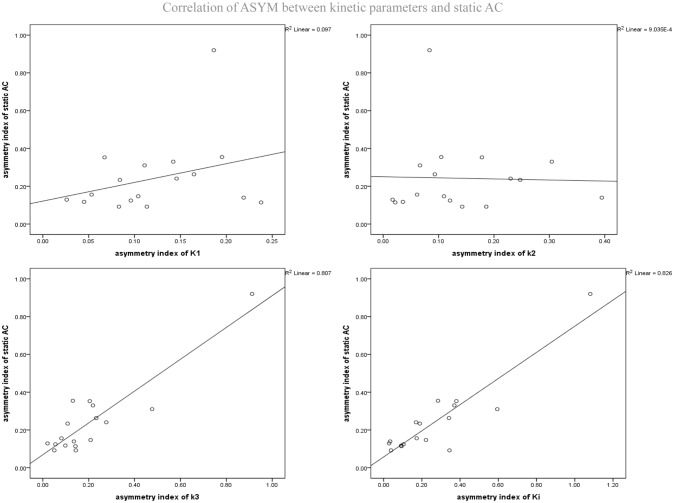
Correlation of ASYM between dynamic FDG PET kinetic parameters and static FDG PET activity concentration (AC) in patients with epilepsy. ASYM of static AC positively correlated with ASYM of *k*_3_ and *K*_i_ (spearman correlation coefficients, 0.60, 0.70, respectively; both *P* < 0.05).

As mentioned above, the different dynamic parameters represent the different processes and steps of glucose metabolism into brain neurons, while static AC represents the basic state of glucose metabolism in the cerebral cortex. Dynamic parameters are related to static AC but different, by calculating the correlation of ASYM dynamic FDG PET kinetic parameters and static FDG PET AC in patients, it may be known that the low metabolic state of epileptic foci is most correlated with which dynamic parameter, which may provide insight understanding for the hypometabolism at epileptic foci. ASYM of static AC positively correlated with ASYM of *k*_3_ and *K*_i_ (spearman correlation coefficients, 0.60, 0.70, respectively; both *P* < 0.05) (Table 2 and Figure 6). Glucose net metabolic flux *K*_i_ is a comprehensive parameter, so we can preliminarily speculate that the hypometabolism of epileptic foci is related to *k*_3_.

**Table 2:** Correlation between ASYM of static AC and ASYM of kinetic parameters.

	*K*_1_	*k*_2_	*k*_3_	*K*_i_	Static AC
*K*_1_ (ASYM)	–	0.24	0.46	0.11	0.26
*k*_2_ (ASYM)	0.24	–	0.14	0.09	0.12
*k*_3_ (ASYM)	0.46	0.14	–	0.75^b^	0.60^a^
*K*_i_ (ASYM)	0.11	0.09	0.75^b^	–	0.70^b^
static AC (ASYM)	0.26	0.16	0.60^a^	0.70^b^	–

*Kinetic parameters: influx (K_1_), efflux (k_2_), phosphorylation (k_3_) and net metabolic flux (K_i_), AC, activity concentration. ASYM, asymmetry indexes. (^a^P < 0.05, ^b^P < 0.01).*

## Discussion

In this small epilepsy cohort study, we had assessed whether kinetic parameters derived from dynamic FDG PET have value for identifying epileptic foci by using static AC from static FDG PET as a reference. What’s more, we had examined the effect of blood flow and glucose metabolism separately in the epileptic zone by dynamic and static scans, which is likely to be relevant for uncovering the mechanism of epileptic hypometabolism. To avoid the invasive arterial blood sampling procedure from patients, we obtained IDIF from carotid artery in early frames of the dynamic image, and used IDIF with an irreversible two tissue compartment model to calculate rate constants for FDG influx, efflux, phosphorylation, and net metabolic flux (Zanotti-Fregonara et al., 2011). This methodology is proven to be robust (Hammers et al., 2008; Bertran et al., 2016), relatively simple to apply, and suitable for routine clinical practice. Although input function obtained with arterial blood samples is considered as gold standard, it is difficult to perform in routine clinical studies because it is laborious, risky and uncomfortable for patient. Post scan blood handling and analysis are additional exposure to the research personnel (Bertran et al., 2016). In order to compare across subjects, we calculated ASYM for both static AC and kinetic parameters base on templated-based analysis. By comparing the left and right hemisphere in the same segment within subject, any potential bias resulted from bloodless input function measurements was eliminated (Archambaud et al., 2013).

Our ASYM measurements showed mean reduction in all kinetic parameters, specifically *K*_1_ and *k*_2_ by 12–15% and *k*_3_ and *K*_i_ by 21–27%. These findings were consistent with a prior study of glucose metabolism inside the epileptic foci using arterial blood sample dynamic FDG PET, which reported decrease of *K*_1_, *k*_3_, and *K*_i_ by 18, 25, and 26% (Cornford et al., 1998), respectively, their *k*_2_ was also found decreased (by 13%) but was not statistically significant. As we know, the larger side-by-side view of metabolic asymmetry, the easier it is to identify the hypometabolic epileptogenic regions. It is not uncommon that different interpretations of one single image by visual assessment among different readers occur, and subtle metabolic abnormalities of epilepsy might not be identified easily (Archambaud et al., 2013). We compared metabolic abnormalities of epilepsy by VOI-based ASYM to alleviate observer bias inherent in the manual procedure, so analyses across subjects are more robust. We found that blood flow indicated by rate constants *K*_1_ and *k*_2_ may be less sensitive to ipsi- and contra-lateral difference (smaller ASYM). ASYM correlation between kinetic parameters and static AC also implied the same trend. Another study (Fink et al., 1996) which measured cerebral blood flow using [15O]butanol found that blood flow in the epileptic foci was unaffected compared to controls. Those findings were also in accordance with evidence of interictal capillary influx/efflux being less reliable for detecting epileptogenic zones previously reported by PET and SPECT (Fink et al., 1996; Nelissen et al., 2006). Our results also showed that there were no statistical differences in ASYM between net metabolic flux (*K*_i_) or the phosphorylation (*k*_3_) and static AC, which demonstrated that *K*_i_ and *k*_3_ from dynamic FDG PET without arterial blood sampling can also accurately localize the epileptogenic zone in this small cohort of the drug-resistant epilepsy patients.

It is noteworthy that biggest reduction was in the *K*_i_ (mean ASYM = 0.27), though these changes were not significantly different from static AC (mean ASYM = 0.24), and the results shown the greatest correlation between static AC and *K*_i_ (spearman correlation coefficients, 0.70, *P* < 0.01). However, there are some differences between the two measurements. *K*_i_ reflects only the uptake rate of FDG which is metabolized while static AC measurement also includes the unmetabolized fraction of FDG (Freedman et al., 2003). It was proposed that there might be lower background intensity in *K*_i_ maps (Wanet et al., 2011) while the higher background intensity in static AC images. This feature will be helpful when using the *K*_i_ map for locating epileptic foci. Our evaluation for detecting epileptic foci by using side-to-side ASYM can offset signal-to-background ratio difference. In this condition it may be difficult to get the differences between *K*_i_ and static AC. It may show significance when the sample size and regional classification increase. Therefore, *K*_i_ map may have more potential in surgical planning because boundaries mapped with *K*_i_ can be more clear-cut, the delineation can be small and more accurate (Visser et al., 2008; Wanet et al., 2011). Larger studies with *K*_i_ map evaluation are warranted.

Perhaps the most important point of our study was that using dynamic FDG PET scan allows us to investigate the different effects leading to interictal hypometabolism within epileptogenic focus. Our finding of reduction in glucose utilization was higher than reduction in influx/efflux with statistical significance suggested that phosphorylation was more affected than reduced blood flow in the foci area. Traditionally, seizures are caused by hyper-excitable state and excessive discharge of a population of neurons. However, mounting recent evidence indicates that the glucose metabolism is an important regulator of neuronal and network excitability (Bazzigaluppi et al., 2017). Studies demonstrated that hypometabolism can induce neural network suppression unstable. together with improving brain neuronal glucose metabolism can control seizures (Freeman et al., 2009; Nei et al., 2014), suggested that the hypometabolism may be the cause of the epileptic process. Many factors can affect cerebral glucose metabolism, such as blood flow, key enzymes in glucose metabolism pathways. In epileptic model and patients, abnormal capillary vasodynamics are mediated by mural cell constrictions which is complex and changeable (Leal-Campanario et al., 2017). While blood flow contribute to epilepsy is controversial (Tewolde et al., 2015), we found that FDG uptake in the epileptic foci was minimally affected by influx and efflux of FDG.

The majority of energy required by the brain is provided by the oxidation of glucose via glycolysis and the tricarboxylic acid (TCA) cycle. It has been proven 38–52% of all refractory epilepsy patients showed a reduction in seizure frequency under ketogenic diet (Nei et al., 2014), ketone bodies generating acetyl-coenzyme-A which enters the TCA cycle directly, hence supporting ATP production in the absence of glucose. Nerve cells can enhance the TCA cycle when extracellular glucose concentration decreased as well as glycolysis partly inhibited (Malkov et al., 2018). So, we can speculate that reduced glucose utilization in epileptogenic focus is underlain by glycolytic pathway inhibition. Previous studies have shown down-regulated glycolytic molecules in mouse model of epilepsy (McDonald and Borges, 2017; McDonald et al., 2017). We demonstrated that phosphorylation of glycolytic pathway by rate constant k_3_ had more impact in epileptogenic focus. This suggested that seizure-reduced glucose phosphorylation might contribute to interictal hypometabolism. Overall this work has revealed new potential targets for new drug design, if new therapeutic agents can improve glucose phosphorylation within epileptogenic focus, it may cure seizures.

There were several limitations in the study. First, manual delineation of ROIs without MRI fusion for the carotid might introduce inter- and intra-observer variability. By keeping a rather constant ROI size at the same brain level, we believe the inter-observer variability will be minimized. Second, the lack of blood sampling hindered the absolute quantification of blood input, making a direct comparison of kinetic parameters across subjects not possible. The use of ASYM which is calculated within subject alleviates the need of blood quantification. Clinically, the use of IDIF for dynamic FDG scans and a side-to-side ASYM comparison of kinetic parameters are not only informative but also provides an alternative to the invasive blood sampling for quantitation. Third, a common template-based analysis such as AAL always cannot achieve a perfect match between VOIs and the underlying anatomic structure of individual subjects due to mis-registration and anatomic variability. This more or less will impact the ASYM, impeding the ability of detecting epileptic foci especially if the area or change between ipsi- and contra-lateral side is small. The problem can be improved by delineating ROIs from the individual subject’s MRI such as the approach in FreeSurfer (Kemppainen et al., 2018) which of course, depends heavily on the quality of MRI scan and the accuracy of brain segmentation and parcellation tools. Another limitation of our study was the number of patients, we have tried to include patients with epileptic foci in various brain regions to mitigate the problem.

## Conclusion

Compared to static FDG PET scan, dynamic FDG PET can provide equally effective and complementary measure for epileptogenic zone detection in this small cohort. Kinetic parameters allow one to understand the pathophysiology of hypometabolism within the epileptogenic focus. Our results showed that interictal epilepsy was more impacted by glucose phosphorylation than by capillary influx. The hypometabolism of epileptic foci may be related to reduced glucose phosphorylation. Therefore, improving glucose phosphorylation may be the focus for future therapeutic drug development to treat epilepsy.

## Author Contributions

YT designed the method, acquisition of data, and prepared the manuscript. J-SL, SH, and BT designed the method, aided in data analysis, revised and approved the manuscript. ZZ, JL, TL, and YL aided in data acquisition and interpretation.

## Conflict of Interest Statement

The authors declare that the research was conducted in the absence of any commercial or financial relationships that could be construed as a potential conflict of interest.
